# The functionality variation among health facility governing committees under direct health facility financing in Tanzania

**DOI:** 10.1371/journal.pgph.0000366

**Published:** 2022-05-19

**Authors:** Anosisye Mwandulusya Kesale, Christopher Mahonge, Mikidadi Muhanga

**Affiliations:** 1 Department of Policy Planning and Management, Sokoine University of Agriculture, Morogoro, Tanzania; 2 Department of Development Studies, Sokoine University of Agriculture, Morogoro, Tanzania; University of Ottawa, CANADA

## Abstract

Decentralization reforms through Direct Health Facilities Financing (DHFF) have empowered Health Facility Governing Committees (HFGCs) to participate in different governance aspects to improve service delivery at the facility level. However, there is little research on how empowered HFGCs perform in the context of the DHFF. The purpose of this study was to evaluate the functionality of HFGCs under DHFF in Tanzanian primary health care facilities that had variation of performance in 2018. To collect both qualitative and quantitative data, the study used a cross-section design. The study had a sample size of 280 respondents, who were chosen using a multistage cluster sampling technique from 32 primary health care facilities that were practicing DHFF. Data was collected via a closed-ended structured questionnaire, in-depth interviews with chairpersons of HFGCs, and Focus Group Discussions. To examine the functionality of HFGCs, researchers used descriptive and theme analysis. In the 2018-star rating assessment, the study discovered that HFGCs functioned well in both high and low-performing health facilities. When HFGCs from high-performing health facilities were compared to HFGCs from low-performing health facilities, it was discovered that HFGCs from the high-performing health facilities had comparatively high functionality. The functionality of HFGCs in Tanzania has thus been impacted by the DHFF context.

## 1. Introduction

Improving the health service delivery is a precondition for achieving Universal Health Coverage in Lower- and Middle-Income Countries (LMICs) [1, 2]. The LMICs have embarked on health sector reforms through Decentralization by Devolution to enhance the improvement of health service delivery at the primary health care [3, 4]. Decentralization by Devolution (D by D) entails “the transfer of governance responsibility/decision-making powers for specified functions to sub-national levels through publicly or privately owned institutions that are largely outside the direct control of the central government” [5]. The D-by-D have created community governance structures such as Health Facility Governing Committees (HFGCs) to allow communities participation in the governance of the primary health care facilities [6, 7]. The HFGCs are responsible for planning, implementing and controlling service delivery at the primary health facilities to bring about health systems responsiveness, increased efficiency and effectiveness and increase accountability to health service providers [8, 9]. Therefore, functional HFGCs promise meaningful community participation in the health service delivery at primary health care. In the context of this study, the functionality of HFGC is defined as the ability of HFGCs to accomplish their devolved functions in their health facilities.

Community participation in the planning, implementation and monitoring of health service delivery at the primary health facility the widely recognized for influencing the efficiency, accountability and responsiveness of service providers. The established HFGCs as governance organs in the primary health care facilities representing the community are appreciated for their contribution in shaping health service delivery [10]. This is because HFGCs provide opportunities for individuals to participate in making decisions affecting their health and they are answerable for their performance [11, 12]. HFGCs are charged with specific functions to accomplish in the governance process such as participating in planning [1, 13, 14], managing finances [15] procurement process [13], addressing communities health challenges [14, 16] and managing health workers [17]. Through accomplishing their devolved functions, HFGCs are expected to rise the health facility performance and improve health service delivery at the primary health care [18–20] however empirical evidence suggests that HFGCs performance in developing countries is low and below the expectation [21]. Some cited reasons for the limited performance of HFGCs are limited decentralization to HFGCs, members not being able to know their responsibilities, shortage of funds in their facilities, compositions of HFGCs and educational level [18, 22]. To address HFGCs functionality challenges developing countries have embarked on fiscal decentralization to empower HFGCs and health facilities to accomplish their responsibilities [23, 24].

In Tanzania, HFGCs were established in 1999 as part of decentralization reforms to increase community involvement in the administration and management of primary health care facilities [4, 25]. According to the HFGCs establishment and operationalization guidelines of 2013, HFGC are composed of eight (8) members in the dispensaries and nine (9) in health centers [26]. The members of the committees are community representatives, health facility in charge, local government representatives, private health services representatives and faith-based representatives. Community representatives are elected by the community while other representatives either are appointed by their groups or by virtual of their positions [26]. The following functions are delegated from these HFGCs: Participate in the development of facility plans and budgets for the management of facility income, expenditures, and performance. Similarly, to gather funds for construction and maintenance management. Furthermore, discussing and addressing the community’s concerns, as well as rallying the community to participate in the improved Health Community Fund.

Tanzania like other LMICs introduced HFGCs in 199 embarked on fiscal decentralization through Direct Health Facility Financing (DHFF) approach since 2017/18 [26]. The DHFF approach involves direct depositing of facility finance from different sources such as finance from the Ministry of Finances (MoF) to the health facility accounts. DHFF also empowers HFGCs with planning and budgeting, financial management, procurement and other governance powers and autonomy to the HFGCs [5, 8]. Before the introduction of DHFF, health facilities and HFGCs had no fiscal powers and autonomy over the health facility resources because the council’s levels were ultimately controllers of facility finances and were also planning and budgeting for health facilities [25, 27]. These practices led to delay in disbursement of finances to the facility which caused the late implementation of health intervention as well as minimized the functionality of HFGCs. However, there is limited information on how the empowered HFGCs perform to accomplish their assigned powers and functions in primary health facilities after the introduction of the DHFF arrangement in Tanzania. The few studies which have been conducted on DHFF have just concentrated on assessing the influence of DHFF on health services delivery in general [5, 8, 25, 27, 28]. This study embarked on assessing the functionality of HFGCs in primary health facilities implementing DHFF in Tanzania.

## 2. Methods

### Study area

The research was carried out in four geographical regions, each of which has different geographical council that were categorized based on their performance (high performance and low performance regions) in the star rating assessment conducted in January 2018, the same year that the DHFF was launched in Tanzania. The President’s Office Regional Administration and Local Government undertook a star rating evaluation to evaluate the performance of Tanzania’s basic health care institutions. The star rating assigned stars to facilities based on their performance, with star 0, 1,2,3,4,5 being assigned to the facilities. Primary health care facilities that received three stars or more were considered excellent performers, while those that received less than three stars were considered bad performers. As a result, Mbeya and Kilimanjaro were chosen because the majority of their health facilities performed well, while Ruvuma and Songwe were chosen because the majority of their health facilities performed poorly. The HFGCs at both high- and low-performing health institutions were studied to see if the DHFF setup had an impact on their ability to carry out their devolved functions. This is because the literature suggests that health facility performance is strongly associated with HFGC performance. Therefore, when health facility performance is high, HFGC performance tends to be high as well, and vice versa [19, 25].

### Research design

In this study, a cross-sectional design was used. Between February and April 2021, quantitative and qualitative data were collected from a large number of subjects or respondents at a single point in time to analyze the functionality variation among HFGCs in chosen health institutions.

#### Sample size and sampling procedure

Select regions, councils, primary health facilities, and members of HFGCs were sampled using a multistage cluster sampling technique. The first stage began with the identification of regions with a predominance of high-performing health facilities (Mbeya and Kilimanjaro) and low-performing health facilities (Ruvuma and Songwe). The second stage was to choose a council from the selected region that had a high-performance rate for the majority of health facilities (Chunya, Siha, Madaba District Council, and Tunduma Town Council) and a council with a low performance rate (Mbozi district council, Mbeya city council, Songea, and Moshi Municipal). Stage three entailed selecting four primary health care facilities from each of the councils chosen in the previous stage. The health facility selection was divided into two categories: the type of facility (health center or dispensary) and the facility’s performance. Stage four involved selecting respondents who are members of HFGCs, with proportional sampling used to choose at least 9 members from each HFGC, resulting in a total of 280 respondents. Table 1 provide a summary of the sampling process, techniques and inclusion criteria involved in this study.

**Table 1:** Sampling process and sampling techniques.

Stage	Respondent	Sampling procedure	Inclusion criteria
First stage	Four (4) regions selected Kilimanjaro, Mbeya, Ruvuma and Songwe	• purposive	Performance of the region, Zonal representation
Second Stage	8 LGAs selected; Two LGAs from each region selected in stage one	• purposive	Performance of the LGAs in star rating assessment, nature of the LGA (Urban and Rural)
Stage Three	32 health facilities were selected from all (8) councils. 2 health centers and 2 dispensaries from each LGA because they all implement DHFF	• Stratification of health facilities into Health centers and Dispensaries • Purposive selection of health centers and dispensaries	Performance of health facility (A good and poor performing health center and dispensary), Location of the facility within the LGA (Diversity)
Stage Four	280 HFGC members; 9 members from each selected health facility	• Simple random selection of HFGC members	members of the HFGC

### Data collection method

To collect quantitative data from each HFGC member, a closed-ended structured questionnaire focused on specific HFGC functions was used. The data gathering software (database) was built using Open Data Kit (ODK). All of the data was then entered into the ODK. To collect data, a quantitative approach called mobile data collection (MDC) was used. Mobile phones were used to collect data, which was subsequently sent to a central server. Four research assistants went through a three-day training program on mobile data collection skills and techniques, which was followed by skill pre-testing in facilities outside of the study area. The gathered data was sent to the researcher using the ODK platform. As part of quality control, all of the facilities were given GPS coordinates, therefore all of the research assistants used GPS-enabled tablets. The poll received 280 responses out of a total of 288 HFGCs.

#### Qualitative data collection

In-depth interviews and Focus Group Discussions were both used to acquire qualitative data (FGDs). A total of 14 in-depth interviews with HFGC Chairpersons from various health facilities were undertaken to learn about the HFGC members’ experiences performing their devolved functions under the DHFF. A total of 14 Focus Group Discussions (FGDs) with 6 to 9 participants were also held, involving all members of the HFGCs. After achieving saturation, the number of interviews and focus groups was reduced. Interviews and FDGs were held in specially designed rooms where participants were free to speak freely without being interrupted.

### Quantitative analysis

Analysis was done using Statistical Product and Service Solution (SPSS) statistical software (version 25) and Statistical Analysis System (SAS version 9.4). Descriptive and inferential statistics were used to analyze data, Frequency tables were used to describe the sample and the characteristics of the participants. The dependent variable for this study was the functionality of HFGC. The Functionality of HFGCs in improving health service delivery under the DHFF context was statistically analyzed based on the experience of HFGC members in accomplishing their assigned functions as indicated in the four points Likert Scale in which each point was in percentage. Then, the four points Likert scales were dichotomized for further analysis. The first two points namely “Very Low” and “Low” were coded 0 and “High” and “Very High” were coded 1. the score of functionalities was calculated by summing up all dichotomized variables. The possible minimum score was 0 and the possible maximum score was 9. The functionality score was categorized into two categories those who scored above the median (5) were regarded as good functioning while those who scored 5 or less were regarded as bad functioning. This practice is consistent with the analysis conducted in the study of health system responsiveness conducted in Tanzania [29]. The independent variables for this study included nine (9) items (functions) which determined the functionality of HFGCs as shown in Tables 2 and 3.

**Table 2:** Demographic characteristics of HFGC members.

Variable	Frequency	Percent
**Region**		
Kilimanjaro	93	33.21
Mbeya	64	22.86
Songwe	54	19.29
Ruvuma	69	24.64
**Type of Health Facility**		
Dispensary	161	57.50
Health center	119	42.50
**Position**		
Chairperson	43	15.36
Secretary or facility in charge	34	12.14
Member of the HFGC	203	72.50
**Age**		
<30	32	11.43
31–45	100	35.71
46–60	107	38.21
61+	41	14.64
**Sex**		
Male	139	49.64
Female	141	50.36
**Education level**		
Primary	150	53.57
Secondary	64	22.86
Certificate	24	8.57
Diploma	30	10.71
Advanced diploma	5	1.79
University degree	7	2.50

**Table 3:** HFGCs functioning under DHFF in primary health facilities that had high performance as per 2018 star rating assessment n = 146.

Independent Variable	Dependent Very low n (%)	Variables Low n (%)	High n (%)	Very high n (%)	Mean (SD)
Participate in Preparing facility plan and Budget according to community needs	7 (5.22)	18(13.43)	82(61.19)	27(20.15)	3.78(1.08)
Managing facility income and expenditure	7(5.22)	28(20.90)	66(49.25)	33(24.63)	3.67(1.21)
Participate in managing the procurement of health equipment, drugs and services	6(4.48)	14(10.45)	78(58.21)	36(26.87)	3.93(1.05)
Participate in managing facility performance	10(7.46)	35(26.12)	67(50.00)	22(16.42	3.42(1.25)
Management of facility resources	5(3.73)	29(21.64	71(52.99)	29(21.64)	3.67(1.15)
Mobilization of facility finances from different sources	10(7.46)	45(33.58)	60(44.78)	19(14.18)	3.25(1.27)
Participate in managing constructing facility infrastructures	7(5.22)	29(21.64)	75(55.97)	23(17.16)	3.58(1.16)
Discussing the challenges confronting the community	5(3.73)	26(19.40)	73(54.48)	30(22.39)	3.72(1.13)
Mobilizing community to join improved Health Community Fund	4(2.99)	15(11.19)	56(41.79)	59(44.03)	4.13(1.07)

### Qualitative data analysis

Qualitative data was analyzed through the adoption of five steps analysis process. The process was designed to accommodate patterns and themes to be captured emerged during data collection. The collected data were recorded through audio therefore analysis process started with transcriptions of the audio into notes from in-depth interviews and FGDs. Then the data were coded based on keywords relating to the functionality of HFGCs to capture the variations and commonalities among categories of primary health facilities. Then thematic areas relating to the objective of the study and which also related to the guiding framework of the study were identified to help to explain the data and their relationship.

#### Variables of the study

The functionality of HFGC was the study’s dependent variable. The functionality of HFGCs in primary health care facilities implementing DHFF was statistically analyzed based on the experience of HFGC members in carrying out their assigned responsibilities, as expressed on a four-point Likert Scale with percentages for each point. The four-point Likert scales were then dichotomized for additional investigation. The first two points, "Very Low" and "Low," were given a 0 rating, while "High" and "Very High" were given a 1. The functionality score was derived by adding all dichotomized factors together. The lowest possible score was 0 and the highest possible score was 9. The functionality score was divided into two categories: good functioning and poor functioning. Those who scored over the median (5) were considered good functioning, and those who scored 5 or below were considered poor functioning. This practice is in line with the findings of a Tanzanian study on the responsiveness of the health system. As shown in Table 3, the independent variables for this study included nine (9) components (functions) that determined the functionality of HFGCs. This functions are assigned to the HFGCs by the Tanzania HFGC establishment and operationalization guideline of 2013 [26].

### Ethics approval and consent to participate

The Sokoine University of Agriculture provided the IRB with the number SUA/ADM/R. 1/8/668. The permit was then filed to the President’s Office Regional Administration and Local Government (PO-RALG) of Tanzania to be granted permission to conduct research on local government authorities. PO-RALG issued a permit with the registration number AB.307/323/01 to allow the study to be conducted in the chosen areas. All human participants in this study provided written informed consent by signing consent forms before being included in the investigation.

## 3. Results

### Social demographic characteristics

The social demographic characteristics of this study involved locations, types of health facilities, positions of members of HFGCs, age in terms of the number of years of members of HFGCs, sex of members of HFGC and education level of members of HFGCs. More details have been indicated in Table 2.

### The functionality of HFGCs under DHFF context

Table 3 indicates the functionality of HFGCs in primary health facilities that had high performance in 2018 when DHFF started to be implemented in all public primary health facilities in Tanzania. the functionality of HFGCs in Table 3 is indicated through the functionality of HFGCs in their devolved functions in primary health facilities.

Table 4 indicates the functionality of HFGCs in various aspects devolved to them in primary health facilities that had low performance in 2018. The functionality of HFGCs was evaluated through the Likert Scale level. Each function was measured by using the mean score.

**Table 4:** HFGCs functioning under DHFF in primary health facilities that had low performance as per 2018 star rating assessment n = 134.

Independent Variable	Dependent Very low n (%)	Variables Low n (%)	High n (%)	Very high n (%)	Mean (SD)
Participate in Preparing facility plan and Budget according to community needs	2(1.37)	29(19.89)	77(52.74)	38(26.03)	3.82(1.07)
Managing facility income and expenditure	6(4.11)	22(15.07)	76(52.05)	42(28.77)	3.86(1.12)
Participate in managing the procurement of health equipment, drugs and services	5(3.42)	17(11.64)	91(62.33)	33(22.60)	3.89(1.00)
Participate in managing facility performance	8(5.48)	27(18.49)	78(53.42)	33(22.60)	3.69(1.17)
Management of facility resources	4(2.74)	25(17.12)	77(52.74)	40(27.40)	3.85(1.09)
Mobilization of facility finances from different sources	8(5.48)	55(37.67)	63(43.15)	20(13.70)	3.22(1.24)
Participate in managing constructing facility infrastructures	5(3.42)	29(19.86)	86(58.90)	26(17.81)	3.68(1.09)
Discussing the challenges confronting the community	3(20.5)	18(12.33)	89(60.96)	36(24.66)	3.94(0.96)
Mobilizing community to join improved Health Community Fund	1(0.68)	16(10.96)	69(47.26)	60(41.10)	4.17(0.94)

**Fig 1:**
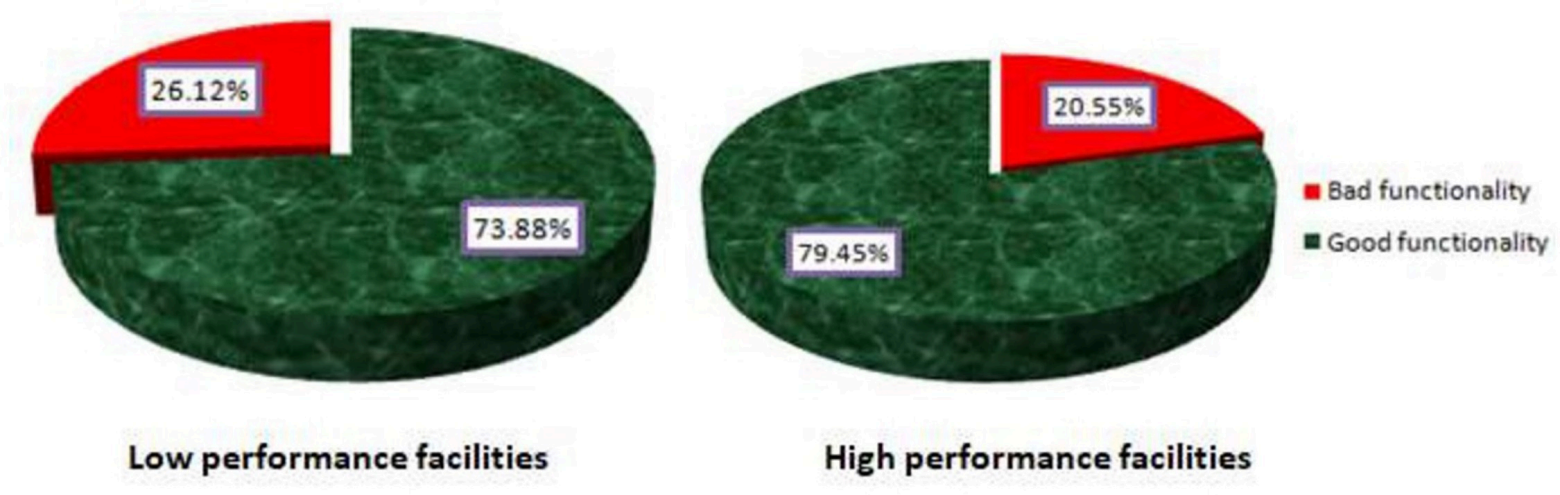
Functioning level of HFGCs in primary health facilities under DHFF implementation.

The results in Fig 1 indicate that HFGCs from both primary health facilities that had high and low performance in 2018 have good functionality. HFGCs from primary health facilities that had high performance have recorded 79% of good functionality while the counterpart has recorded 73% of good functionality Under DHFF implementation.

**Fig 2:**
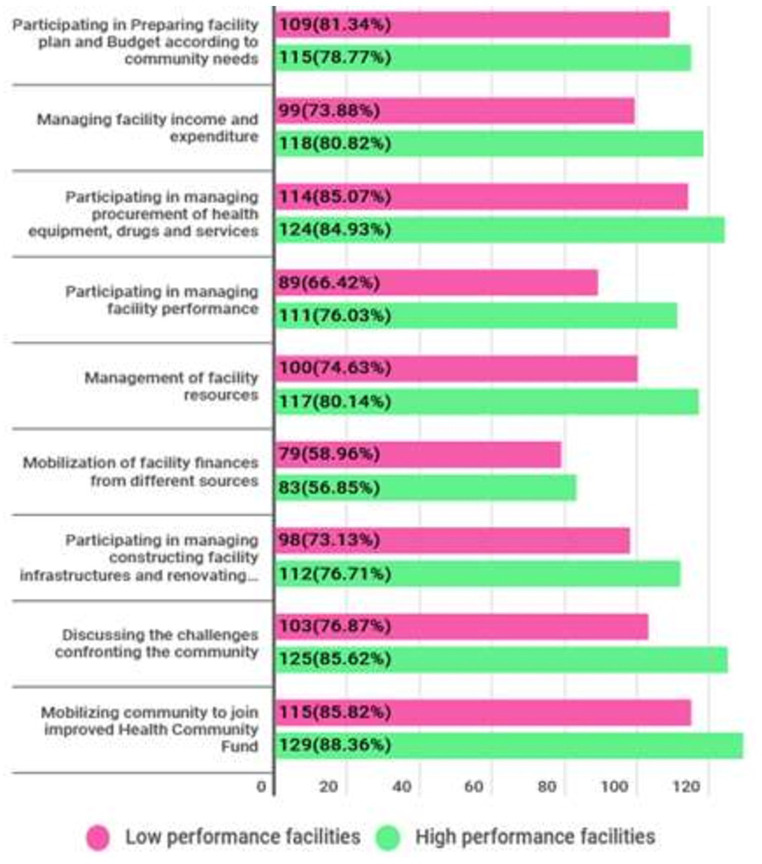
Prevalence of functionality HFGCs in their devolved functions.

The result in Fig 2 indicates the current performance of HFGCs from both health facilities that had high and low performance. Quantitatively, the results show that HFGCs from both health facilities which had high and low performance are relatively functioning good in participating in mobilizing the community to join improved community health funds (CHF), participating in procurement of health commodities, medicines and other services, participating in the planning and budgeting as well as discussing community challenges.

### Perspectives of HFGC members on the functionality of HFGCs under DHFF implementation

#### Management of facility resources

The participants in a primary health facility that had high performance in the start rating assessment conducted in 2018 reacted that they have been participating in the management of facility resources in the health facility as one of their assigned functions.

*“We make sure to take care of all our resources not to get damaged or been stolen*, *but this is for few observable resources and not all the resources because for me I don’t know how many resources the facility has”*

#### Collecting and addressing community health challenges

The major aim of establishing of HFGCs was to ensure the community forms part in addressing community health challenges because HFGCs members are from the community so they have adequate knowledge of the community health challenges. Participants from both interviews and FGDs agreed that they have been linking community and health facilities even though they have not been able to address all the challenges.

*“To sincerely we still have problems here which are very difficult to be solved by our levels such as availability of medicines*, *medical commodities and health workers”*

#### Mobilizing community to join Community Health Fund (CHF)

Participants highlighted that they have hardly been participating in mobilizing community members to join CHF for the purposive of mobilizing facility funds and also helping community members to be able to get health services. Different mechanisms of mobilizing the community have been mentioned by the participants.

*“Yes*, *we are still doing that and mobilize them to join the CHF*, *using ten cell leaders based on where they live and through that*, *we get many people*. *even when there is an open meeting on the street*, *we also influence them”*

#### Financial management

Facility intervention to the large extent requires finance to smoothly be implemented. HFGCs are assigned the role of overseeing the utilization and management of facility funds. HFGCs members perceive that DHFF has granted them powers to exercise such a role. Participants explained the extent they have been participating in managing such funds.

*“Ok*, *we understand the system*, *when the money arrives*, *we are informed by the accountant or the secretary of the committee*, *so we are responsible to know how much money we have received and call a meeting with the committee ready to facilitate the plans as scheduled”*.

#### Participate in planning and budgeting

Participants also explained how they have been engaging in the preparation of health facility plans and budgets in their areas.

“*We are part of the planning team and indeed we get the opportunity to place our ideas and community challenges when the facility plan and budget is tabled to our HFGCs for approval”*

### Perspectives of HFGCs members on their functionality from primary health facilities that had low performance in 2018

#### Participating in preparing comprehensive health facility plan and budget

Members of HFGCs perceive that they participate in the planning process and have been able to channel community issues in the plan and budgeting. They also recognized that they have been overseeing the implementation of the plan. some other members thought that last year they have not participated in the preparation of the plan

*“About the comprehensive plan*, *first we prepare the plan*, *we sit here as a team because we were given the paper for plan generation and were directed that we must include specialists*, *teachers and the committee together with the facility workers and we fill the tables accordingly*, *and this is the first step”*

### Mobilizing communities to join community health fund

The reaction of the HFGCs on their functioning on issues relating to the mobilization of the community to join community health funds was very positive. Through both interviews and FGDs members perceive that they have done enough but shortage of medicine in many health facilities.

*“We go and visit the societies try to educate people on a certain thing*, *for example*, *people were not used to visiting our health facility for different services so the committee tends to educate them and influence them to use our facility*, *also to join the CHF”*

### Management of health facility finances

Health services delivery requires finances; therefore, mobilization and management of facility funds is vert imperative for improving primary health care delivery. the participant responded on their experience in the mobilization and management of funds.

*“We have had powers and freedom in managing facility finances now since we know what health facility has and that money cannot be used without our authorization*. *So*, *we feel responsible for mobilizing funds and we have been doing so through approaching different stakeholders”*

Another responded

*“As the council construction agent present the budget which was a high amount and the society did not have so we reached an agreement that we will join force with the council and the money collected will be used for that”*.

### Participation in the procurement of medicines, medical commodities and other materials

In this theme, participants responded that since the beginning of the implementation of DHFF they have been part of the procurement process of their health facility. They outlined their roles such as approving the demands of items to be procured, aligning procurement with facility plan and budget and being part of the team, which confirms the items which are being delivered to the health facility. Also highlighted some challenges.

*“Maybe the problem is interference from the council level because sometimes we were told that the council has already identified someone who is going to renovate and build our buildings so it became difficult for us to supervisor the council tenderer*”

### Addressing community health challenges

Participants revealed that since they are members elected by the communities, therefore, they have been collecting community health challenges through different mechanisms and through HFGC meetings they discuss to address those challenges

*“For example*, *now we know that medicines on the facility are the result of the presence of money*, *in order to have more medicine you must have money*, *so you come to find out on the absence of the medicine in the facility even the iCHF card won’t be of much help”*

## 4. Discussion

The study looked at how well HFGCs functioned in a DHFF environment in primary health care facilities that had a range of performance in 2018. The goal was to see if there was any variation or similarity in HFGC functionality under DHFF among a group of HFGCs from primary health care facilities with varying performance. According to the findings of this study, the functionality of HFGCs from both low and high performing primary health facilities in 2018 is generally good, as HFGCs from primary health facilities with varying performance have recorded good functionality above 70% under DHFF, as shown in Fig 1. In a 2018-star rating assessment, the average HFGC functionality across the country was less than 60%. Indeed, qualitative data from interviews and focus groups have revealed that HFGCs are more involved in their devolved functions as a result of DHFF implementation. The findings of this study are similar to those of earlier studies conducted in Tanzania to analyze the impact of DHFF in primary health care institutions, which revealed that DHFF led to enhanced community ownership, more autonomy, and improved financial management [8, 30].

However, there is a difference in functionality among HFGCs, with HFGCs from primary health facilities that had a high-performance during star rating assessment in 2018 having relatively good functionality, scoring 79.45 percent (see Fig 1), compared to HFGCs from primary health facilities that had a low performance, scoring 73.88 percent (see Fig 1). The functionality of HFGCs has improved under the DHFF setup, according to qualitative findings, because HFGCs have been able to fully participate in many areas that represent their functions. That means that in the context of DHFF implementation, HFGCs have felt empowered to apply their powers and hold health service providers accountable. HFGC’s performance in supervising and monitoring health service providers increased as a result of fiscal decentralization in primary health care through a different arrangement, such as Direct Facility Financing (DFF) in Kenya [29, 31, 32]. Fiscal decentralization, according to the literature, increases the functionality of service providers and provides autonomy to different sub-national entities, such as HFGCs, when done properly in different situations [33–35].

It was discovered that HFGCs from both high and low-performing primary health institutions have good functioning in similar duties such as rallying the community to join CHF, engaging in the planning and budgeting process, facility procurement, and discussing community concerns (see Fig 2). This means that the implementation of the DHFF in primary health care institutions has broadened the scope and offered possibilities for HFGCs to carry out their allocated responsibilities as outlined in the empowerment framework [33]. The precise areas in which HFGCs have been found to have strong functionality are the most strategic tasks that determine facility performance, so good functionality of HFGCs in those areas can help primary health facilities improve to some extent. According to McCoy et la, the functionality of primary health care facilities’ HFGCs determines the performance of health facility whether they operate well or poorly [19].

HFGCs from high-performing primary health facilities, on the other hand, were found to be relatively effective in some specific duties, such as encouraging communities to join CHFs, discussing community concerns, managing facility resources, and monitoring facility income and expenditure. While HFGCs from primary health care facilities that performed poorly in 2018 were discovered to have reasonably strong functionality in mobilizing funds from other sources and participating in planning and budgeting. The findings imply that HFGCs from high-performing health facilities work well in a variety of specific roles, which explains why their overall performance is superior to that of their counterparts. This confirms the argument provided by McCoy *et al* and Kessy that the performance of health facilities tends to be reflected in the performance of HFGCs [4, 19].

Finally, it was discovered that implementing DHFF in primary health care facilities increased the functionality of HFGCs in many delegated functions. Even while there is no direct statistical evidence of a direct association between HFGCs and DHFF, the evidence from the 2018 Star rating assessment supports our position. The average performance of HFGCs according to the star rating assessment conducted prior to the implementation of DHFF was below 60%, but this study found that the performance of HFGCs in selected primary health facilities implementing HFGCs is above 70% in both low and high performing primary health facilities. Participants believe that DHFF has given them with a conducive environment in which to do their tasks.

## 5. Conclusion

The study discovered that HFGCs functioned well in both high and low-performing health institutions in the 2018-star rating assessment, the same year the DHFF began. In comparison, HFGCs from high-performing health facilities were discovered to have relatively high performance. According to the findings, HFGCs from both high and low performing health facilities have good functionality in similar activities but have comparatively poor functionality in a few devolved functions such as mobilizing resources from other sources and managing facility performance. These findings suggest that increasing the functionality of community governance systems in primary health care institutions in developing countries will necessitate extensive budgetary decentralization. Fiscal decentralization through a different arrangement, such as the DHFF, allows other types of decentralization, such as political decentralization, to be meaningful, as this study found that HFGCs increased their functionality in various aspects, such as addressing community health challenges. When community governance structures (HFGCs) are financially empowered, they are better able to oversee health care provider accountability and improve PHC health service provision.
